# Prostatic adenocarcinoma with transitional cell features among the Saudi Arabian population: a registry review

**DOI:** 10.1097/MS9.0000000000000123

**Published:** 2023-01-18

**Authors:** Yahya Ghazwani, Belal N. Sabbah, Mohammad A. Algahfees, Tarek Z. Arabi, Abdulrahman K. Alageel, Ziyad F. Musalli, Ebtesam S. Almutairi, Ahmed Alasker

**Affiliations:** a College of Medicine, King Saud University; b Department of Urology, King Abdulaziz Medical City; c Department of Medicine, King Abdullah International Medical Research Center; d College of Medicine, Alfaisal University; e College of Medicine, King Saud Bin Abdulaziz University for Health Sciences, Riyadh, Saudi Arabia

**Keywords:** prostate cancer, prostatic adenocarcinoma, Saudi Arabia, transitional cell

## Abstract

Prostate cancer is the third leading cause of death from cancer among American men. Acinar adenocarcinoma is the most common form of prostate cancer; however, there are several nonacinar adenocarcinoma variants, such as transitional cell carcinoma of the prostate and ductal adenocarcinoma.

**Materials and Methods:**

A retrospective cohort study was conducted on all Saudi patients diagnosed with adenocarcinoma of the prostate with transitional cell features. The data was collected from the Saudi Cancer Registry, which collects tumor data from all private, military, and Health Ministry hospitals in Saudi Arabia through five regional offices.

**Results:**

Out of 3608 patients, only 16 (0.44%) had adenocarcinoma with transitional cell features. All the tumors under investigation were malignant and constituted. Only 6.2% of the tumors were well-differentiated, 43.8% were moderately differentiated, and 50.0% were poorly differentiated. Among the included patients, 56.3% of the patients (*n*=9) died. There were no significant factors associated with death among patients, including the demographic and tumor-related variables.

**Conclusion:**

To the authors’ knowledge, this is the first study describing the prevalence of adenocarcinoma with transitional cell features and its characteristics in Saudi Arabia. The authors have demonstrated that this rare subtype may be more prevalent than what was originally believed. It is necessary for future studies to assess the effectiveness of various treatment modalities to combat it. Furthermore, identifying risk factors – if any – may be crucial in the prevention of its development among men worldwide.

HIGHLIGHTS

- Acinar adenocarcinoma is the most common form of prostate cancer; however, there are several nonacinar adenocarcinoma variants, such as transitional cell carcinoma of the prostate and ductal adenocarcinoma.
- It is demonstrated that this rare subtype may be more prevalent than what was originally believed.
- Identifying risk factors – if any – may be crucial in the prevention of the development of this cancer among men worldwide.

Prostate cancer is the third leading cause of cancer-related deaths in American men^1^. The most common type of prostate cancer is acinar adenocarcinoma; however, there are several nonacinar adenocarcinoma variants, including transitional cell carcinoma of the prostate (TPCC) and ductal adenocarcinoma (DAC)^2^. TPCC is an aggressive malignant tumor with an average survival of 17–29 months after diagnosis^2^,^3^. TPCC is characterized by a single or stratified layer of cuboidal cells with pleomorphism of the nuclei^4^. TPCC is almost always diagnosed in association with urethral or bladder transitional cell carcinoma, with primary TPCC being extremely rare. The incidence rate of TPCC among 11,678 patients was 1.1% of all prostatic neoplasms^2^.

Distinguishing between adenocarcinoma with transitional cell features and common subtypes, such as acinar adenocarcinoma has many prognostic and therapeutic implications. Establishing the correct diagnosis will prevent the potential initiation of inappropriate hormone therapy that might occur if the tumor were to be misconstrued as a variant of acinar – type prostatic adenocarcinoma. Mai *et al*.^5^ first reported six cases of prostatic adenocarcinoma with intraductal or invasive components displaying TPCC in 2002. Since then, there have been limited data regarding this unique type of prostatic cancer and its prognostic implications. Hence, in this study, we evaluated type prostatic adenocarcinoma cases and their survival times using the Saudi Cancer Registry (SCR). To the best of the authors’ knowledge, this is the first study to describe this subtype in Saudi Arabia.

## Materials and methods

This retrospective cohort study included all Saudi patients diagnosed with primary adenocarcinoma of the prostate with transitional cell features from 1 January 2008 to 31 December 2018. Patients who were diagnosed with tumors that metastasized to the prostate or were non-Saudi were excluded from the study. The data was collected from the SCR, which collects tumor data from all private, military, and Health Ministry hospitals in Saudi Arabia through five regional offices. The variables were grouped according to gender, age, marital status, region tumor behavior, tumor grade, tumor extent, the basis of the diagnosis, and the mortality status on last contact.

Frequencies and percentages were used to express categorical variables, and median and interquartile ranges were employed to present patients’ ages as numerical variables. Factors associated with death were assessed using a Wilcoxon rank sum test for age and a Fisher’s exact test for other categorical variables. Survival analysis was performed by depicting a Kaplan–Meir curve, computing the median survival time, and the 1-year survival rate. Statistical analysis was implemented using RStudio (4.1.1), and a *P* value of less than 0.05 indicated statistical significance. This study has been registered on the Research Registry under the unique identifying number (UIN) research registry 8505. This study is reported in compliance with the Strengthening The Reporting Of Cohort Studies in Surgery (STROCSS) criteria^6^.

## Results

### Demographic characteristics of patients

Out of 3608 prostate cancer patients, 16 (0.44%) had adenocarcinoma of the prostate with transitional cell features. The median (interquartile range) age of patients was 67.0 years (61.2, 71.2). Less than half of them were residing in the Western region (43.8%) and the Central region (37.5%). The majority of patients were married (75.0%). Furthermore, more than half of them were managed in hospital under the Ministry of Health (56.2%) as shown in Table 1.

**Table 1:** Demographic characteristics of patients.

Parameter	Category	*N* (%)
Age (y)	Median (IQR)	67.0 (61.2, 71.2)
Age category	<30	1 (6.2)
	30 to <45	0
	45 to <60	3 (18.8)
	60 or more	12 (75.0)
Marital status	Single	2 (12.5)
	Married	12 (75.0)
	Unknown	2 (12.5)
Region	Central	6 (37.5)
	Western	7 (43.8)
	Southern	2 (12.5)
	Northern	1 (6.2)
Hospital type	Ministry of Health	9 (56.2)
	Military	4 (25.0)
	Private	3 (18.7)

IQR, interquartile range.

### Characteristics of tumors

All the tumors under investigation were malignant and constituted adenocarcinoma with transitional cell features. Only 6.2% of the tumors were well-differentiated, 43.8% were moderately differentiated, and 50.0% were poorly differentiated. Tumors were localized in 37.5% of the patients and distant metastasis was reported among 31.2% of them. The remaining patients had no known status of tumor localization. Most of the tumors were diagnosed based on the histological testing of the primary lesions among the majority of patients (75.0%) as shown in Table 2.

**Table 2:** Characteristics of tumors.

Parameter	Category	*N* (%)
Diagnosis method	Histology of primary	12 (75.0)
	Histology of metastases	1 (6.2)
	Cytology/hematological	1 (6.2)
	Death certificate only	2 (12.5)
Behavior	Benign	0
	Malignant	16 (100.0)
Grade	Grade I (well differentiation)	1 (6.2)
	Grade II (moderate differentiation)	7 (43.8)
	Grade III (poor differentiation)	8 (50.0)
Extension	Localized	6 (37.5)
	Distant metastasis	5 (31.2)
	Unknown	5 (31.2)

### Survival analysis and factors associated with death

**Figure 1:**
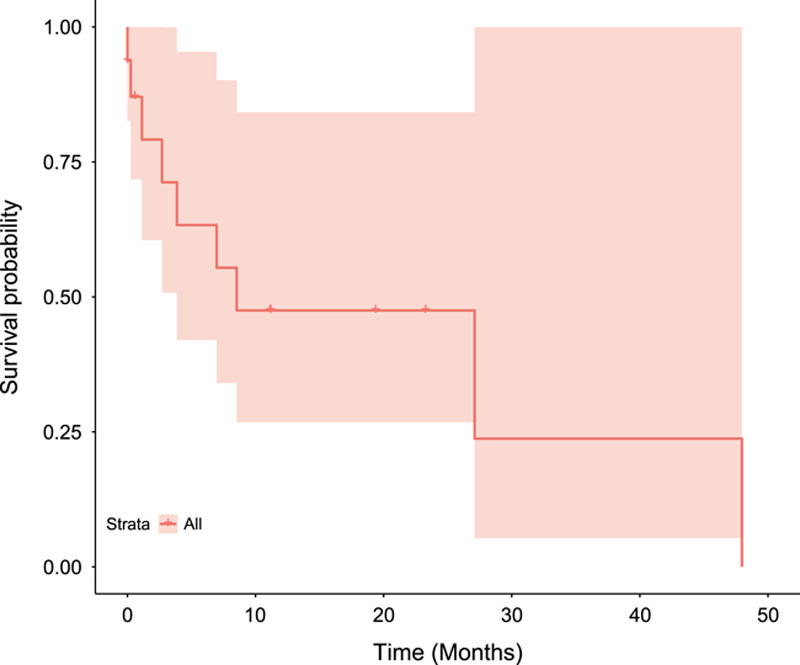
Kaplan−Meier curve which depicts censored data for patients with prostate cancer.

Among the included patients, 56.3% of the patients (*n*=9) died. As shown in Figure 1, the median survival time was 8.53 months (95% CI: 3.87–13.11), and the 1-year survival rate was 47.5% (95% CI: 26.8–84.2). There were no significant factors associated with death among patients, including the demographic and tumor-related variables as shown in Table 3.

**Table 3:** Factors associated with death among patients with prostate cancer.

		Death, *N* (%)		
Parameter	Category	No (*N*=7)	Yes (*N*=9)	*P* value
Age	Numerical	67.0 (60.5, 68.5)	67.0 (63.0, 75.0)	0.457
Region	Central	2 (28.6)	4 (44.4)	0.443
	Western	3 (42.9)	4 (44.4)	
	Southern	2 (28.6)	0	
	Northern	0	1 (11.1)	
Hospital type	Ministry of Health	4 (57.1)	5 (55.6)	0.868
	Military	1 (14.3)	3 (33.3)	
	Private	1 (28.6)	1 (11.1)	
Marital status	Single	0 (0.0)	2 (25.0)	0.473
	Married	6 (100.0)	6 (75.0)	
Grade	Grade I (well differentiation)	1 (14.3)	0	0.443
	Grade II (moderate differentiation)	2 (28.6)	5 (55.6)	
	Grade III (poor differentiation)	4 (57.1)	4 (44.4)	
Extension	Localized	3 (60.0)	3 (50.0)	>0.999
	Distant metastasis	2 (40.0)	3 (50.0)	
Diagnosis method	Histology of primary	6 (85.7)	6 (66.7)	0.475
	Histology of metastases	1 (14.3)	0 (0.0)	
	Cytology/hematological	0 (0.0)	1 (11.1)	
	Death certificate only	0 (0.0)	2 (22.2)	

## Discussion

Adenocarcinoma of the prostate with transitional cell features is a rare entity that is not often encountered in clinical practice. Thus, a large gap regarding this topic exists within the literature, especially in the Middle East. In this registry review, we assess the demographic and tumor-related characteristics along with the survival time among patients diagnosed with this rare entity. Saudi Arabia is unique ground for its high prevalence of risk factors, such as being located in the Gulf region which has a highly endogamous population^7^.

Prostatic adenocarcinoma with transitional cell features was first described in 2002 by Mai *et al*.^5^ after they evaluated radical prostatectomy specimens and surgical pathology files in Canada. The authors found a 1-year survival rate of 66.67%, and all patients died within 3 years of diagnosis. In our present study, we identified 16 patients with transitional cell carcinoma with a 1-year survival of 47.5%. Although the 1-year survival rate in our patient population is lower, only 56.3% have died, and the difference may be linked to our larger sample size. In comparison, studies have demonstrated that primary prostatic transitional carcinoma and metastatic DAC have a 5-year survival of 52 and 72%, respectively^8^,^9^. Hence, adenocarcinomas with transitional cell features present with a prominently worse prognosis and 1-year survival rate compared to other subtypes of prostatic cancer.

Differentiating between areas expressing features of DAC and those expressing features of TPCC is crucial to the diagnosis. Histologically, the TPCC component is discerned by abundant cytoplasm, nuclear pleomorphism, and the absence of glands^5^. Immunohistochemistry may also be a useful tool in distinguishing between both components. Immunostaining for prostate-specific antigen (PSA) shows moderate to strong expression in the DAC components^5^,^10^. Kunju *et al*.^11^ assessed the staining of PSA in prostatic adenocarcinoma and TPCC. The authors found that PSA stained strongly in 95% of adenocarcinomas versus 0% of TPCC cases. Furthermore, it has been demonstrated that thrombomodulin (TM) stains positively in various types to transitional cell carcinomas and fails to stain in prostatic adenocarcinomas^12^. Accordingly, the TPCC components of adenocarcinoma with transitional cell features are reactive to TM staining and DAC components remain nonreactive^5^. It is also worth noting that areas expressing TM demonstrate less reactivity to PSA staining^5^. Interestingly, TPCC components show weak to moderate reactivity to cytokeratin 7, a common marker of prostatic transitional cell carcinoma^5^,^10^.

Between 2003 and 2017, the incidence of metastatic prostatic cancer was 5% among US men^13^. Compared to other subtypes, it has been demonstrated that adenocarcinoma subtypes displaying transitional cell features present with a high risk of distant metastasis. Among six patients previously described in the literature, all patients developed metastasis to the bone, five to lymph nodes, three to the liver, two to the brain, and one to the lung^5^. Although distant metastasis was not present among all our patients, the metastasis rate remains relatively high at 31.2%. It is worth noting that the true metastasis rate among our patients may be higher (or lower), as the status of tumor localization was unknown in 31.3% of patients. However, according to the present study and previous literature, it seems that the metastasis rate is higher than that of other neoplastic subtypes.

### Limitations

Our present study faces a couple of limitations. First, the incidence of underreporting of findings to tumor registries may reduce the external validity of our findings. Unfortunately, this is an unavoidable limitation to all studies utilizing cancer registries^14^. It has been hypothesized, however, that the inclusion of prevalent cancers from death certificates may negate the negative effects of underreporting^15^. Second, the SCR fails to report the treatment modalities used in patients, limiting the ability to assess the effectiveness of treatment modalities among the population. Furthermore, the registry lacks data regarding the patients’ exposure to risk factors; hence, we were unable to fully determine whether adenocarcinoma with transitional cell features is associated with any risk factors.

## Conclusion

Adenocarcinoma with transitional cell features is an aggressive subtype of prostatic cancer which displays features of both prostatic adenocarcinoma and transitional cell carcinoma. To the authors’ knowledge, this is the first study describing the prevalence of adenocarcinoma with transitional cell features and its characteristics in Saudi Arabia. We have demonstrated that adenocarcinoma with transitional cell features may be more prevalent than what was originally believed. Similar to the available literature, adenocarcinoma with transitional cell features was associated with low 1-year survival rates and high distant metastasis rates among our patient population. Our study is greatly limited by the unavailability of certain parameters, such as treatment modalities and risk factor exposure, in the SCR. Hence, it is necessary for future studies to assess the effectiveness of various treatment modalities in adenocarcinoma with transitional cell features. Furthermore, identifying risk factors – if any – may be crucial in the prevention of adenocarcinoma with transitional cell features development among men worldwide.

## Ethics approval

IRB approval was waived for this study.

## Patient consent

Not applicable.

## Sources of funding

This study did not receive funding from any source.

## Author contribution

B.N.S., M.A.A., T.Z.A., A.K.A., Z.F.M., E.S.A., A.A., and Y.G. drafted the manuscript. B.N.S., T.Z.A., and Y.G. contributed to reviewing and finalizing the manuscript. All authors reviewed the manuscript for intellectual content and approved the submission.

## Conflicts of interest disclosure

The authors declare no conflict of interest.

## Research registration unique identifying number (UIN)

1. Name of the registry: NA.

2. Unique identifying number or registration ID: NA.

3. Hyperlink to your specific registration (must be publicly accessible and will be checked): NA.

## Guarantor

Abdulrahman K. Alageel, MD.

## Provenance and peer review

Not commissioned, externally peer-reviewed.

## Availability of data and material

Not applicable.

## Code availability

Not applicable.
